# Software-Based Relative Humidity Compensation for Low-Cost Optical PM Sensors Using Machine Learning: A Case Study from Upper Silesia, Poland

**DOI:** 10.3390/s26216718

**Published:** 2026-10-23

**Authors:** Patryk Sitarek, Krzysztof Emerling, Katarzyna Schmidt

**Affiliations:** Faculty of Science and Technology, University of Silesia, 40-007 Katowice, Poland; patryk.sitarek@smcebi.edu.pl (P.S.);

**Keywords:** optical PM sensors, humidity compensation, ML-based PM correction, aerosol hygroscopicity, air quality monitoring, edge AI

## Abstract

High relative humidity (RH>60%) significantly impairs the accuracy of low-cost optical particulate matter (PM) sensors due to hygroscopic aerosol growth and water droplet interference. Physical heating elements evaporate condensed water but introduce substantial power consumption that limits low-power IoT operation. This study evaluates a software-based relative humidity compensation approach designed to mathematically emulate the output of a physically heated optical sensor using an unheated sensor paired with ambient meteorological parameters (*T*, RH, *P*) across 48,860 10-min records in Upper Silesia, Poland. We benchmarked six machine learning algorithms, Polynomial Regression (PR), Decision Trees (DTs), Support Vector Regressor (SVR), Dense Neural Networks (DNNs), and Long Short-Term Memory (LSTM) networks, against a co-located, heated SDS011 target sensor across both predictive accuracy and full computational profiling (tuning time, training duration, and inference latency). Static non-linear models achieved superior performance, with sixth-degree PR, SVR and DNN reaching $R^{2}=94\%$, $\mathrm{MAE}=1.41–1.61\;\mu g/m^{3}$, and $\mathrm{RMSE}=3.69–3.79\;\mu g/m^{3}$ for ${\mathrm{PM}}_{2.5}$, whereas complex LSTM networks performed substantially worse ($R^{2}=88\%$, $\mathrm{MAE}=2.56\;\mu g/m^{3}$, $\mathrm{RMSE}=5.33\;\mu g/m^{3}$). Computational profiling revealed that static models require negligible inference latencies (0.0002–0.0011ms/sample) and low tuning times (0.004s/fit for Decision Trees), whereas LSTM incurs an inference delay of 0.1215ms/sample (>500× slower) and heavy tuning overhead (148.96s/fit). These findings demonstrate that instantaneous non-linear relationships dominate humidity artifacts, confirming that lightweight software compensation can emulate the effect of hardware heaters on resource-constrained microcontrollers without deep learning overhead.

## 1. Introduction

Low-cost particulate matter (PM) sensors based on optical light-scattering principles, such as the Nova Fitness SDS011, have gained widespread adoption for dense urban air quality monitoring due to their affordability and ease of integration into Internet of Things (IoT) platforms [1,2,3,4]. However, their physical measurement accuracy is severely compromised by ambient relative humidity (*RH*) interference and particle hygroscopic growth [1,5,6,7]. Most work has shown that *RH* starts to significantly impact low-cost PM sensors when it exceeds 80–85% [2,8,9]. For some other units the impact may start lower at 65–70% [10] or even at 50% *RH* [11]. To mitigate humidity-induced measurement errors, researchers have pursued two primary strategies: physical aerosol drying or heating, and computational machine learning (ML) calibration. Physical pre-conditioning using low-cost thermal dryers or resistive heaters has been demonstrated to effectively evaporate water droplets and maintain sample *RH* below salt efflorescence points, significantly improving alignment with reference instruments during high-humidity and fog conditions [1,12]. Concurrently, a broad range of data-driven calibration models has been investigated to post-process raw sensor readings using ambient weather parameters [1,5,7,13,14,15,16,17,18,19,20]. These computational approaches span classical linear and Polynomial Regression methods [3,7,15,16,19,20,21,22,23,24,25], Decision Trees and ensemble algorithms such as Random Forest, XGBoost, and Extra Trees [3,4,5,7,13,15,16,17,18,19,20,21,22,23,25,26,27], Support Vector Machines [3,16,17,21,22,23], neural networks including Multilayer Perceptron and Long Short-Term Memory (LSTM) networks [7,16,17,19,20], causality-driven feature selection [16,28], and multi-model stacking ensembles [18,20,27]. A comprehensive review summarizing the studied low-cost sensors, reference instrumentation, calibration algorithms, and statistical performance metrics across 20 recent studies is detailed in Table A1 in Appendix A. Despite these advances, existing research exhibits three critical limitations. First, most machine learning calibration studies rely on co-locating low-cost sensors directly alongside high-cost, federal equivalent method (FEM) regulatory monitoring stations [5,7,13,14,17,22,23,24,26,27]. This dependence makes calibration impractical for widespread network deployments in regions lacking adjacent regulatory infrastructure. Second, the some of published field evaluations rely on short-to-medium field campaigns lasting from a few days to several weeks [22,27], leaving long-term seasonal stability and sensor drift under extended operational conditions under-explored. Third, while the literature routinely reports predictive accuracy metrics such as $R^{2}$, MAE, and RMSE [1,3,5,7,13,14,15,16,17,18,19,20,21,22,24,27], studies rarely quantify the computational overhead—specifically the execution times required for model training, hyperparameter tuning, and per-sample inference latency, which is vital for determining whether a model can run on edge devices or resource-constrained IoT gateways.

### Novelty and Contributions of This Work

To bridge these gaps, this study presents a self-contained calibration framework based on a dual-station setup equipped with low-cost SDS011 sensors and automated heating modules. The primary novelty of our work lies in establishing a node-to-node calibration scheme where two parallel SDS011 sensor stations were deployed, with one station automatically activating a thermal heater whenever ambient relative humidity exceeded 60%. This configuration allows for the direct data-driven calibration of an unheated sensor node toward a physically dried reference node without requiring co-location alongside an expensive regulatory station. Furthermore, we conducted a continuous field campaign spanning 15 consecutive months, capturing full multi-season weather dynamics, extreme fog events, and long-term sensor stability. Finally, we systematically benchmarked six diverse machine learning models, Polynomial Regression, Decision Tree, Support Vector Regressor (SVR), Dense Neural Network (DNN), and Long Short-Term Memory (LSTM) networks, evaluating not only their statistical calibration accuracy ($R^{2}$, MAE, RMSE) but also quantifying their exact computational execution times for training, hyperparameter tuning, and per-sample prediction latency to evaluate suitability for real-time edge computing.

## 2. Materials and Methods

### 2.1. Sensor Operating Principle and Its Weaknesses

The sensor examined in this study consists of a shaded chamber housing a light source (laser beam) and a light-sensitive diode. A fan ensures uniform airflow within the chamber. The device measures the scattering of laser light to count the number of particles within a given time interval and determine their size, assuming a specific refractive index for the operating conditions. Based on the particle diameter, the mass can be estimated by assuming a standard density for the particle material and that the particles are spherical. By applying this algorithm and considering the frequency of specific particle masses, the mass information can be converted into dust concentration values.

However, this measurement principle applies equally to solid, dry particles (dust) and liquid particles, such as water droplets that form around the condensation point in a wet aerosol. This can lead to inaccuracies in measurements, particularly under high humidity conditions. Optical particulate matter sensors operating on light-scattering principles are highly susceptible to ambient meteorological conditions; in particular, when ambient relative humidity exceeds a certain value. Environmental particles may contain hygroscopic salts like ammonium sulfate or sodium chloride. Their hygroscopicity is characterized by deliquescence and efflorescence points, displaying hysteresis in the humidogram. Both points are related to particle size (smaller particles tend to absorb water at lower humidity levels) and material composition ratios [29]. Particles with diameters smaller than 0.5 µm are challenging to detect with both amateur and professional sensors. Initially, their low mass minimally impacts measurement error, but under humid conditions, these particles grow in size. Once detectable by simple sensors, they are incorrectly interpreted as particles with significantly higher mass, leading to inflated measurement results.

It is important to distinguish between hardware dehumidification and software compensation. Physical inlet heaters evaporate condensed water and reduce relative humidity inside the measurement chamber to prevent water droplet light scattering. In contrast, software-based compensation uses non-linear machine learning models trained on co-located ambient meteorological inputs (T,RH,P) to correct the humidity-induced overestimation of PM mass concentration, effectively achieving the signal output of a physically dried sensor without hardware energy consumption.

### 2.2. Own Measuring Station and Collection of Data

To obtain the measurement dataset, two identical stations were built, inspired by the Nettigo Air Monitor project [30], with a few modifications. Each station is equipped with the SDS011 particulate matter sensor [31]. The influence of relative humidity on SDS011 sensors has been investigated, for example, in [6,9], demonstrating that *RH* levels exceeding 80% significantly degrade sensor accuracy. The manufacturer specifies in the sensor datasheet that relative humidity above 70% is sufficient to introduce noticeable measurement distortion [31]. In addition to evaluating the impact of relative humidity (*RH*) on measurements, the authors of [6] concluded that the SDS011 sensors demonstrated high unit-to-unit reproducibility and a strong linear correlation with the reference TEOM 1400a analyzer (Rupprecht & Patashnick Co., Albany, NY, USA).

The air intake duct of this sensor is connected to a heater, which consists of the SHT31 sensor [32] and a cermet resistor. This setup is enclosed in an aluminum profile that reflects infrared radiation well. The small volume of the chamber and the airflow near the resistor help reduce humidity without significantly affecting measurement accuracy.

**Figure 1:**
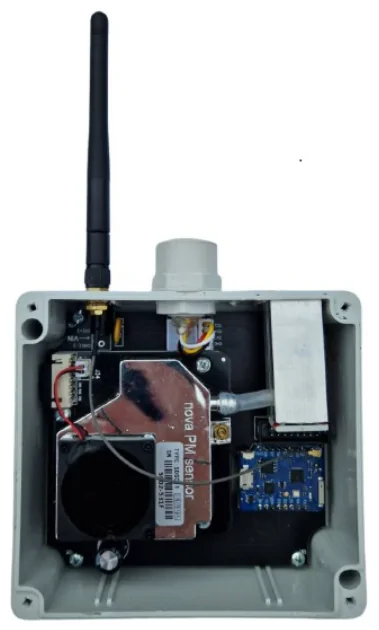
Photo of one of the stations.

Additionally, each station includes an SHT35 temperature and humidity sensor [32] and a BMP180 pressure sensor [33], mounted outside the aluminum profile to isolate them from the air flowing through the PM sensor. The entire setup is connected to a Wemos D1 Mini Pro board [34] with an ESP8266 microcontroller [35]. Figure 1 illustrates the station setup. The sensors are identical in design, with one key difference: one sensor activates the heater when the humidity exceeds 60% (the manufacturer of the SDS011 claims accurate measurements in humidity conditions up to 70%), while the other sensor has the heater permanently deactivated.

The devices operate independently and collect measurements every 3 min. The results are correlated with each other based on timestamp measurements using a nearest-neighbor method, with a maximum allowable difference of 200 s. Records containing missing values were discarded.

PM measurements can be highly variable compared to other parameters (temperature, pressure, humidity). Instantaneous smoke or wind gusts can significantly affect the readings but do not provide reliable information about the overall air pollution levels. To mitigate this, measurements were averaged into 10-min intervals. Additionally, to remove high-frequency noise and sudden non-physical spikes (e.g., localized dust resuspension), a first-order difference *Z*-score filtering technique was applied. The time-series data were chronologically sorted, and consecutive step differences ($\Delta x_{t}=x_{t}-x_{t-1}$) were calculated for PM_2.5_/PM_10_ target variables. These differences were standardized into *Z*-scores ($Z_{t}=\left(\Delta x_{t}-\mu_{\Delta x}\right)/\sigma_{\Delta x}$). Observations exhibiting an absolute step-change *Z*-score greater than 5 ($\left|Z_{t}\right|>5$) were identified as transient outliers and removed from the dataset.

The experimental dataset comprises continuous environmental measurements collected near Lubliniec (Upper Silesia, Poland), a region characterized by elevated PM levels and high relative humidity. Data were gathered from 21 November 2024 to 1 March 2026. Raw sensor readings recorded at 3-min intervals were aggregated into non-overlapping 10-min continuous time steps via arithmetic mean averaging, yielding a total of 48,860 synchronized records.

The dataset includes the following variables:

- Timestamp: Indicates the time of the measurements.
- PM2.5/PM10: The PM_2.5_/PM_10_ value from a sensor with an inactive dryer expressed in micro grams per cubic meter.
- PM2.5/PM10 label: The PM_2.5_/PM_10_ value from a sensor with an active dryer expressed in micro grams per cubic meter.
- Temperature: Air temperature measured in an isolated chamber, expressed in degrees Celsius.
- Humidity: Relative air humidity measured in an isolated chamber, expressed as a percentage.
- Pressure: Atmospheric pressure measured in an isolated chamber, expressed in hectopascals.

Meteorological conditions during the campaign reflected full seasonal climate variations in Southern Poland across the 15-month monitoring window. The ambient temperature averaged 6.56 °C, spanning a wide range from −10.91 °C during winter freezes to 27.36 °C in summer. Mean atmospheric pressure was 992.06hPa. Relative humidity averaged 72.49%, confirming that the majority of measurements occurred under ambient conditions where hygroscopic growth and moisture-induced scattering artifacts (RH>60%) actively affect unheated optical sensor performance.

Table 1 shows the descriptive statistics (mean, standard deviation, minimum, and maximum values) for unheated particulate matter concentrations (PM_2.5_ and PM_10_) alongside ambient meteorological parameters (temperature, relative humidity, and atmospheric pressure) across the 48,860 10-min observation records collected over the 15-month campaign in Upper Silesia, Poland.

### 2.3. Machine Learning Calibration Algorithms

To compensate for relative humidity artifacts, we evaluated five distinct machine learning model families representing a spectrum of structural complexity, ranging from static parametric and non-parametric regressors to temporal deep learning architectures. All static models take a 4-dimensional input feature vector $x_{t}$ $={\left[{\mathrm{PM}}_{\mathrm{unheated},t},T_{t},{\mathrm{RH}}_{t},P_{t}\right]}^{⊤}$ to predict the target heated concentration ${\hat{y}}_{t}={\mathrm{PM}}_{\mathrm{heated},t}$.

Polynomial Regression: Our first approach engaged fitting polynomials to the data using the SciKit-Learn library [36] with a method based on the gradient descent algorithm. Polynomial Regression extends standard Multiple Linear Regression by constructing non-linear feature interactions up to degree *d* [37]. It models the non-linear hygroscopic growth curve by mapping features via a polynomial basis expansion $\phi (x_{t})$. We tested d=1 to d=8 polynomials and observed that increasing the polynomial degree up to d=6 improved the fit, highlighting the importance of considering non-linear relationships in the data to enhance prediction accuracy. However, higher-degree polynomials achieved worse metrics. Therefore, in this paper only the results of polynomial d=6 will be presented.

Decision Tree Regressor (DT): A non-parametric supervised learning algorithm that performs classification or regression by recursively partitioning the feature space into distinct hierarchical branches. At each internal node, the dataset is evaluated against a selected input attribute, splitting the sample into more homogeneous subsets based on impurity reduction metrics such as information gain, the Gini index, or mean squared error [37]. The default Decision Tree regressor from the SciKit-Learn library [38] was used as a first approach and already produced high $R^{2}$ scores for both targets. To further optimize the model, the best hyperparameters were searched for using Successive Halving [39], and the resulting optimal configurations are detailed in Appendix B. The best model obtained differed from the default values, achieving much better performance.

Support Vector Regression (SVR): The extension of Support Vector Machines (SVMs) for regression tasks. While Support Vector Machines (SVMs) construct an optimal hyperplane to maximize the margin of separation between discrete classes in classification tasks, Support Vector Regression (SVR) adapts this principle to continuous target variables by fitting a function within an ϵ-insensitive margin around the data points. For non-linear relationships, kernel functions map the input features into a higher-dimensional space where linear hyperplanes can effectively capture the underlying patterns [40]. The implementation provided in the SciKit-Learn library [41] was used for this purpose. To optimize the SVM regression, algorithms for finding the best hyperparameters were employed (see Appendix B for results). It was noted that searching with a parameter space that was too large did not lead to the best results.

Dense Neural Network (DNN): A Multilayer Perceptron (MLP) consisting of fully connected dense layers with non-linear activation functions. The DNN serves as a universal function approximator, learning hierarchical non-linear combinations of instantaneous meteorological inputs [37]. All neural network models (DNN and LSTM) were constructed and trained using the Keras [42] framework running on top of a TensorFlow [43] backend. The resulting network structure is detailed in Table A2 in Appendix B.

Long Short-Term Memory Network (LSTM): Unlike static models that process each timestamp *t* independently, the recurrent LSTM architecture processes an ordered historical sequence window $X_{t}={\left[x_{t-T+1},\ldots ,x_{t}\right]}^{⊤}$ of length *T* [37]. Through internal memory cells governed by input, forget, and output gating mechanisms, LSTM models potential temporal hysteresis and aerosol conditioning history. The resulting network structure is detailed in Table A2 in Appendix B.

#### 2.3.1. Dataset Partitioning

To prevent data leakage across pollution episodes, a strict chronological split was applied (first 85% training/validation, final 15% independent test set). Static models were trained on instances where RH>60% (40,686 records) to isolate the active humidity correction regime. For LSTM, continuous sequence continuity was maintained during training (T=30). Crucially, all models were evaluated on the exact same chronological test set (RH>60%).

#### 2.3.2. Hyperparameter Tuning

As mentioned before, hyperparameters were systematically optimized: Successive Halving [39] was employed for Decision Trees and SVR, provided by SciKit-Learn library [44]. Successive Halving is an iterative selection process where all candidates (the parameter combinations) are evaluated with a small amount of resources at the first iteration. Only some of these candidates are selected for the next iteration, which will be allocated more resources. For parameter tuning, the resource is typically the number of training samples [44]. Bayesian Optimization [45] via KerasTuner [46] was used for DNN and LSTM architectures. This algorithm gradually learns which regions of the hyperparameter space are most promising by fitting a probabilistic model called a Gaussian process. This allows it to gradually zoom in on the best hyperparameters [37]. For the recurrent LSTM architecture, hyperparameter optimization included systematic tuning of the input sequence length *T*, evaluating historical lookback windows ranging from T=4 to T=50 steps (corresponding to 40 min to approximately 8.3 h of historical context). Across all tested sequence lengths, varying the temporal window did not yield performance improvements. In this paper, the results for T=30 are presented. During the hyperparameter search, overfitting was a common issue. To mitigate this, a callback function for the tuner loop was used to interrupt computing and reject tested hyperparameters if no improvement was achieved for two consecutive epochs. All model architectures can be found in Appendix B.

### 2.4. Technological Stack

All model training, hyperparameter optimization, and performance evaluations were conducted on a dedicated workstation equipped with an 13th Gen Intel(R) Core(TM) i7-13700 processor (24 cores), 15 GB of DDR4 RAM, and an NVIDIA GeForce RTX 2080. The computational environment operated on Linux Mint 21.3 (64-bit). The machine learning pipeline was implemented in Python (version 3.10.12). Classical machine learning models and regression metrics were executed using scikit-learn (v1.2.2), while Deep Learning architectures (LSTM, DNN) were constructed and trained via TensorFlow (2.12.3) with CUDA (v13.0) hardware acceleration.

## 3. Results

**Figure 2:**
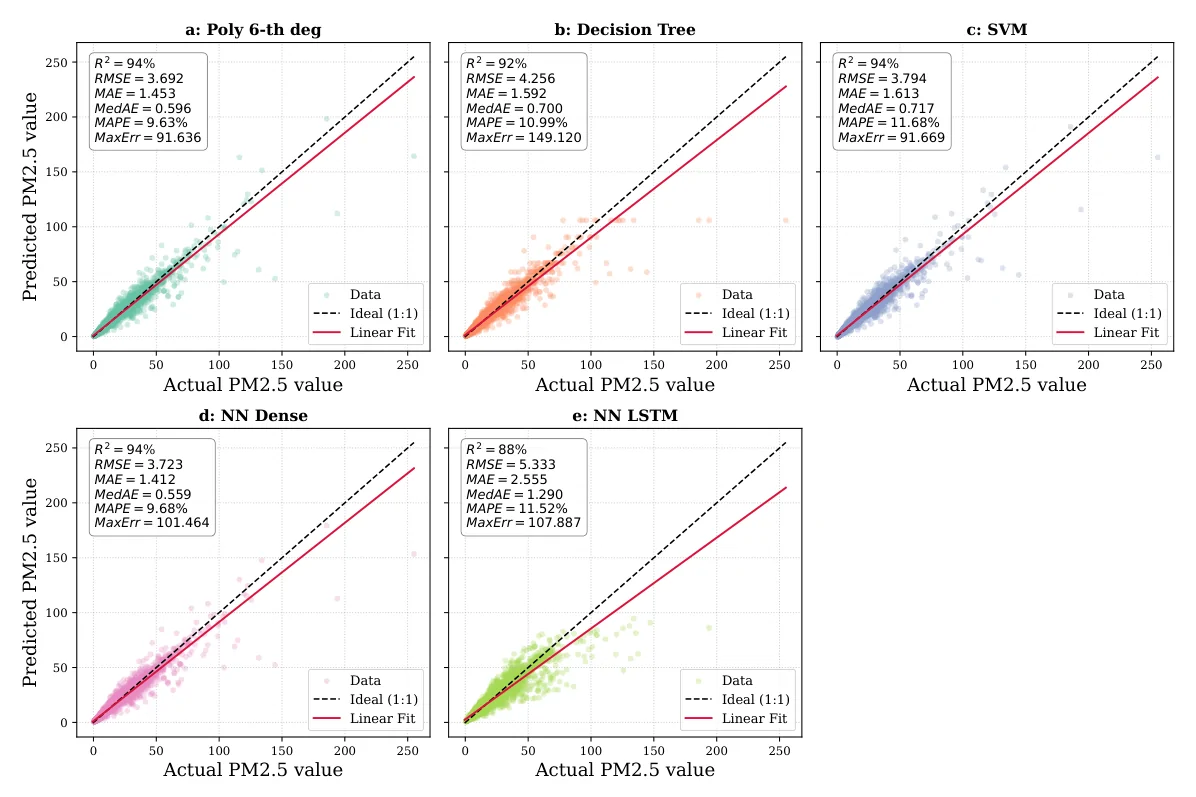
Comparison of predicted versus reference heated PM_2.5_ mass concentrations across evaluated machine learning algorithms:(**a**) Polynomial Regression (d=6), (**b**) Decision Tree, (**c**) Support Vector Machine, (**d**) Dense Neural Network, and (**e**) Recurrent Neural Network. Axes are displayed up to $250\;\mu g/m^{3}$ to maximize visual readability across the primary data distribution (>99.8% of records).

**Figure 3:**
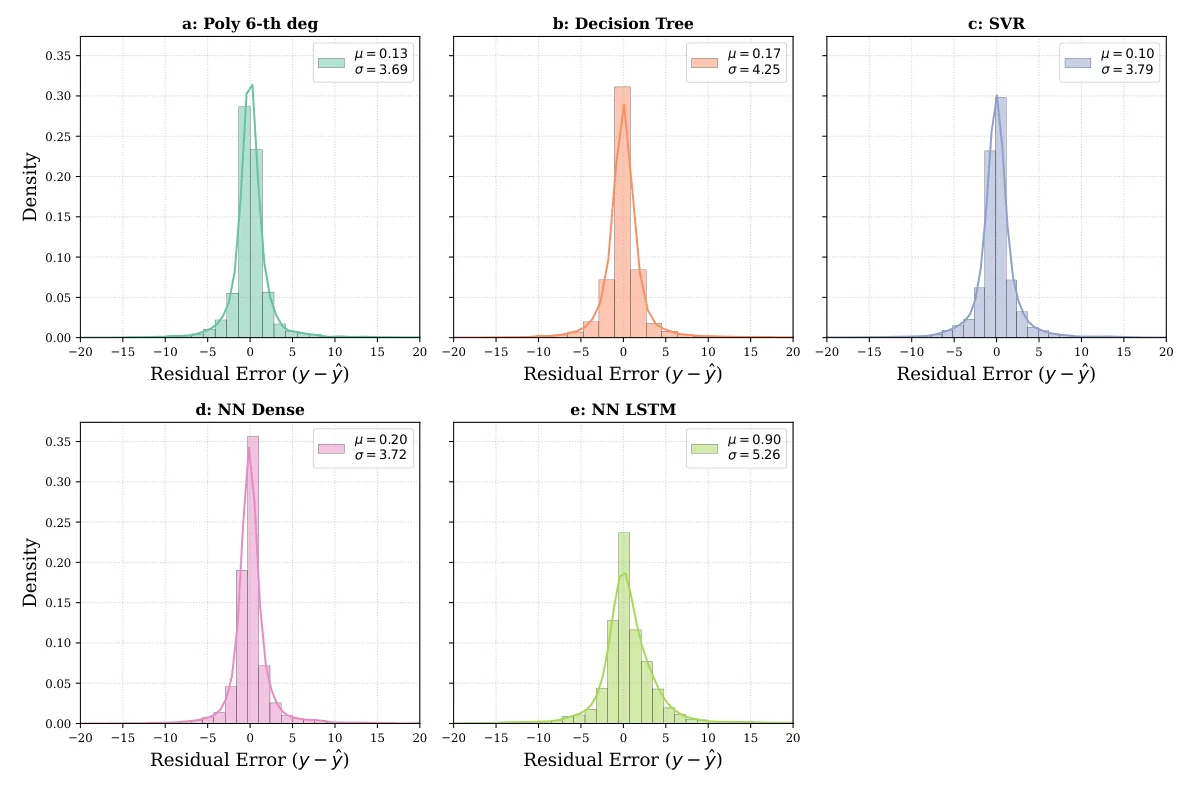
Distribution of PM_2.5_ residual errors for each evaluated model: (**a**) Polynomial Regression (d=6), (**b**) Decision Tree, (**c**) Support Vector Machine, (**d**) Dense Neural Network, and (**e**) Recurrent Neural Network.

**Figure 4:**
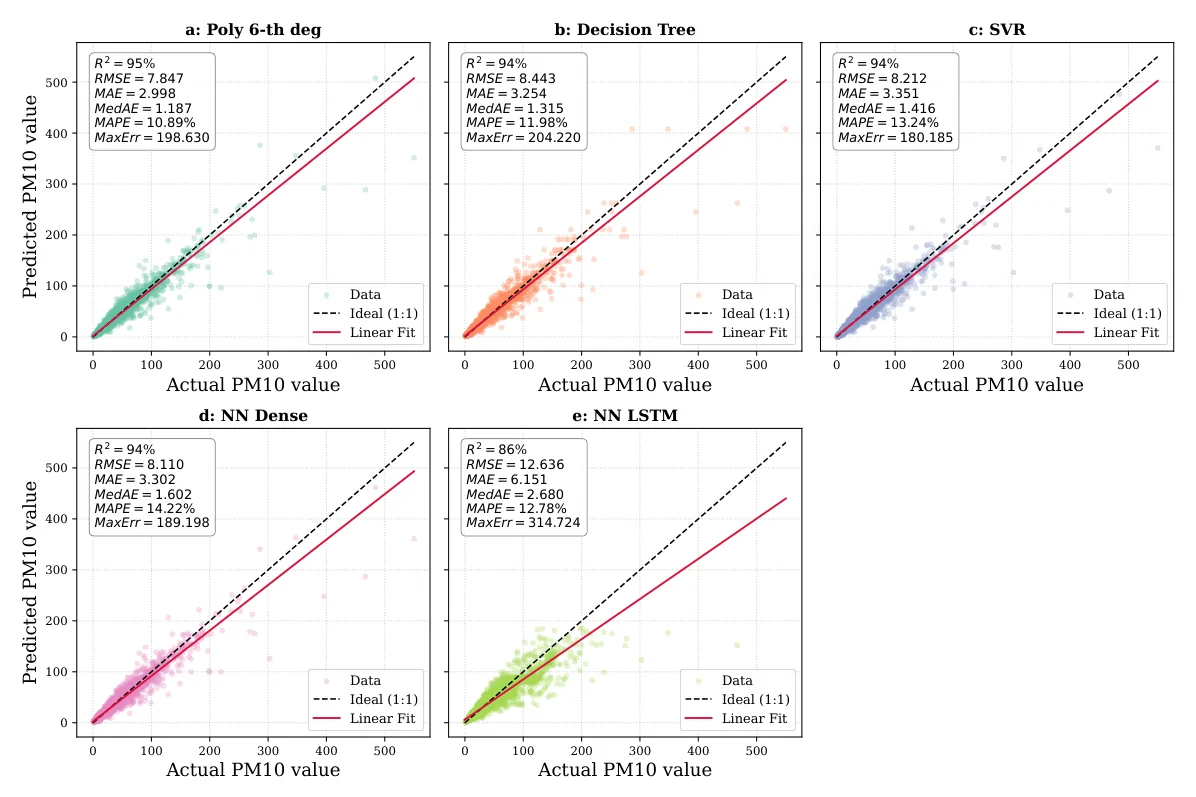
Comparison of predicted versus reference heated PM_10_ mass concentrations across evaluated machine learning algorithms:(**a**) Polynomial Regression (d=6), (**b**) Decision Tree, (**c**) Support Vector Machine, (**d**) Dense Neural Network, and (**e**) Recurrent Neural Network. Axes are displayed up to $500\;\mu g/m^{3}$ to maximize visual readability across the primary data distribution (>99.8% of records).

**Figure 5:**
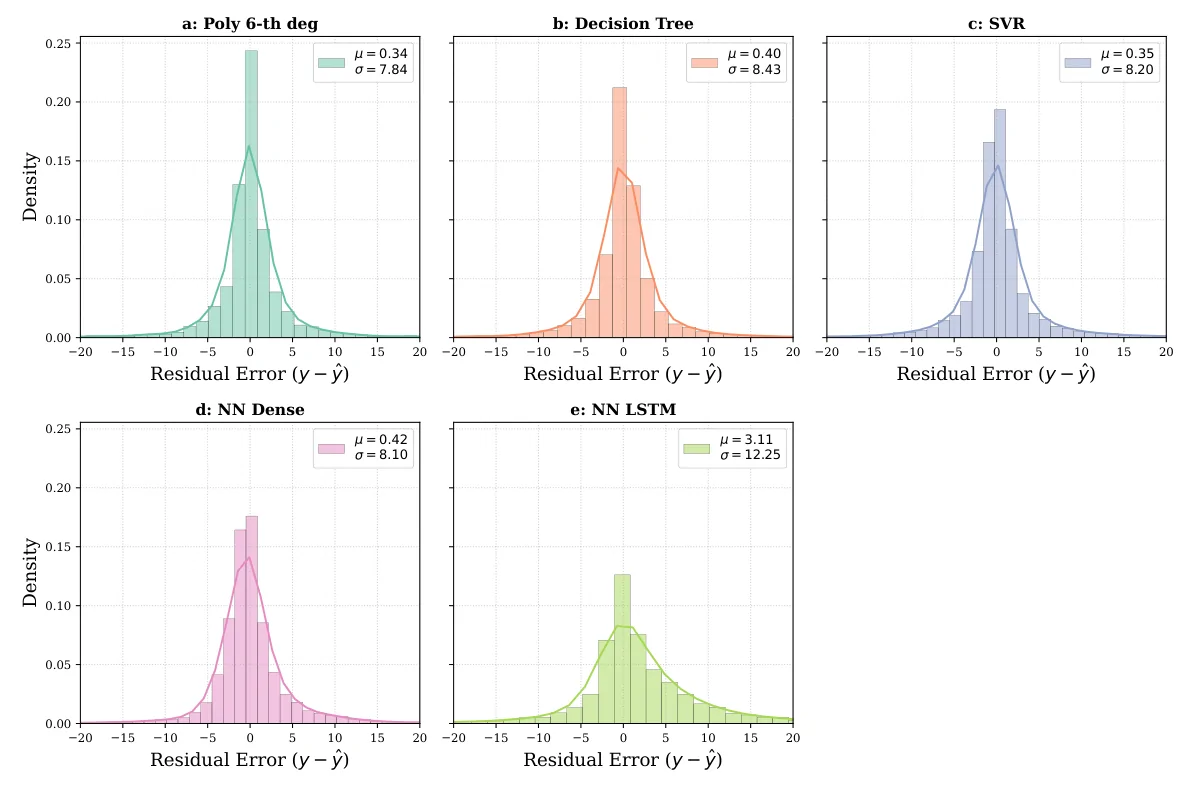
Distribution of PM_10_ residual errors for each evaluated model: (**a**) Polynomial Regression (d=6), (**b**) Decision Tree, (**c**) Support Vector Machine, (**d**) Dense Neural Network, and (**e**) Recurrent Neural Network.

The obtained results for PM_2.5_ and PM_10_ targets are summarized in Figure 2, Figure 3, Figure 4 and Figure 5. Figure 2 and Figure 4 present the predicted versus actual values for each model (a: Polynomial Regression with degree d=6, b: Decision Tree, c: Support Vector Regressor, d: Dense Neural Network, e: Recurrent Neural Network) for PM_2.5_ and PM_10_ respectively. Note that while extreme short-term winter spikes reached a maximum of $383.30\;\mu g/m^{3}$ for PM_2.5_, $942.65\;\mu g/m^{3}$ for PM_10_ (see Table 1), the axis limit in Figure 2 and Figure 4 is displayed up to $250/500\;\mu g/m^{3}$ to optimize graph readability. For each model, the ideal reference line (dashed line) and the actual linear fit (red line) are shown alongside the key regression metrics (R_2_, RMSE, MAE, MSE, MedAE, MAPE, and Max Error). As can be seen, all models, with the exception of the recurrent neural network, achieved similar performance. For the PM_2.5_ target variable, the sixth-degree Polynomial (d=6) Regression model, Support Vector Machine (SVM), and Dense Neural Network (NN) achieved the highest $R^{2}$ scores, with the NN attaining the lowest Mean Absolute Error (MAE). For the PM_10_ target variable, the polynomial (d=6) model yielded the top $R^{2}$ score, closely followed by the Decision Tree (DT), SVR, and NN models. The residual distributions, along with their respective mean and standard deviation values, are illustrated in Figure 3 and Figure 5. The lowest standard deviation was obtained by the NN for the PM_2.5_ target and by the sixth-degree Polynomial Regression for the PM_10_ target. Surprisingly, the recurrent neural network yielded the worst results. This may suggest that while instantaneous local atmospheric states (relative humidity, temperature, and pressure) capture the dominant operational humidity artifacts at 10-min intervals, sub-10-min aerosol dynamics or microphysical hysteresis did not reveal, but may still exist. The practical conclusion remains that static models are far more suitable and computationally efficient for edge deployment.

### 3.1. Comparison of Raw and ML-Corrected Output

**Figure 6:**
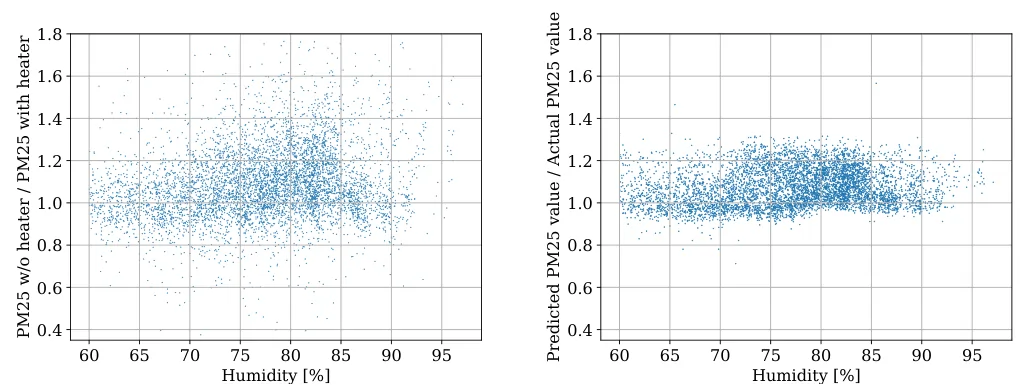
Comparison of PM2.5 concentration ratios versus ambient relative humidity (RH): (**left**) raw ratio of unheated to heated sensor readings (PM_2.5,unheated_/PM_2.5,heated_) showing hygroscopic swelling artifacts above 60%RH; (**right**) ratio of predicted to actual heated concentration (PM_2.5,predicted_/PM_2.5,heated_) after Dense Neural Network compensation, demonstrating tight alignment around 1.0 across the full humidity spectrum (60–98% *RH*).

To evaluate the degree to which machine learning models correct moisture artifacts, the ratio of PM_2.5 *heated*_/PM_2.5 *heated*_ concentrations was analyzed as a function of ambient relative humidity (RH) both prior to and following software compensation (Figure 6). Prior to calibration (Figure 6 left panel), at RH>60%, water condensation and aerosol hygroscopic growth caused significant optical overestimation, pushing unheated-to-heated ratios up to 1.7–1.8 around 80–90% *RH*. Following software compensation using the Dense Neural Network model (Figure 6, right panel), this humidity-dependent overestimation was successfully eliminated. The ratio between predicted concentrations and actual heated target values collapses tightly onto the 1.0 unit line throughout the high-humidity domain (60–98% *RH*), confirming that the algorithm effectively isolates and removes relative humidity artifacts.

### 3.2. Computational Efficiency Analysis

Table 2 presents the execution time for each model. The first column shows the number of tuning fits, followed by the tuning time per fit [s], the training time of the best model [s], and the inference time per sample [ms]. Hyperparameter tuning for Decision Trees was conducted using Successive Halving starting with $n_{\mathrm{candidates}}=1000$, a reduction factor of 3, and 5-fold cross-validation, resulting in a total of 7490 evaluated model fits across seven rounds. For SVR, an initial grid of 250 candidate parameter configurations was evaluated across eight successive reduction rounds (factor 2, 5-fold cross-validation), yielding a total of 494 evaluated fits. Hyperparameter optimization for neural network architectures (DNN/LSTM) was carried out using Bayesian Optimization via KerasTuner across 250 (for DNN) and 100 (for LSTM) distinct trials (max_trials = 250/100, executions_per_trial = 1), with validation MAE (val_mae) serving as the optimization objective. For static models, inference time is measured per individual feature vector. For the LSTM architecture, single-sample inference time corresponds to processing a historical window sequence of length *T* (where T=[30]) to predict the corrected PM concentration for a single time step. The performance times for the PM_10_ target were the same order of magnitude.

## 4. Discussion

The comparative evaluation demonstrates that while raw low-cost optical particulate matter (PM) sensor readings suffer from significant environmental distortion, primarily driven by relative humidity (*RH*) hygroscopic growth effects, machine learning models effectively emulate the effect of a physical heating element by reproducing the readings of a heated sensor. Surprisingly, the highest predictive accuracy ($R^{2}$, MAE) does not monotonically scale with model complexity. Classical models, specifically Polynomial Regression and Support Vector Machine, achieved performance better than deep sequential architectures (LSTM) and comparable to Dense Neural Networks (DNNs). This plateau in performance suggests that for 10-min aggregated ambient PM data, the relationship between meteorological variables (temperature, pressure and relative humidity) and particulate correction is adequately captured by a simple regression model. However, this outcome should not be interpreted as a complete absence of physical aerosol hysteresis, which operate at microphysical scales and may manifest under higher temporal sampling frequencies. Rather, it demonstrates that at 10-min operational scales, instantaneous non-linear relationships dominate humidity distortion, rendering static models (such as sixth-degree Polynomial Regression and Decision Trees) both highly accurate and optimal for low-latency, energy-efficient edge execution.

While predictive metrics offer one perspective, the feasibility of deploying calibration models directly onto edge devices (e.g., microcontroller-based monitoring stations) depends heavily on computational efficiency. Table 2 highlights stark contrasts in operational overhead. Hyperparameter tuning algorithms significantly reduced the search space compared to exhaustive grid searches. However, tuning deep learning models (LSTM, DNN) via Bayesian Optimization required orders of magnitude more time (up to 14,895 s for LSTM across 100 trials) compared to tree-based approaches (≈30 s for 7490 evaluated fits).

Single-sample inference latency represents the critical bottleneck for real-time edge processing. Decision Trees and SVR achieved sub-microsecond prediction times (0.0002 ms to 0.0003 ms per sample), making them ideal candidates for low-power 8-bit or 32-bit microcontrollers (e.g., ESP32) with negligible memory footprints. In contrast, the LSTM network exhibited an inference latency of 0.1215 ms per sample—over 500 times slower than the Decision Tree—while requiring floating-point unit (FPU) hardware and higher VRAM/RAM allocations during deployment.

### Applicability Limits, Model Generalizability and Future Work

While the proposed computational heating emulation method effectively compensates for relative humidity (*RH*) artifacts in low-cost optical PM sensors, the generalizability of the trained empirical parameters must be interpreted within the context of local atmospheric conditions. The magnitude of particle water uptake and hygroscopic growth is strongly dictated by aerosol chemical composition, notably the concentration of deliquescent salts such as ammonium sulfate and sodium chloride, which exhibit distinct deliquescence and efflorescence hysteresis behavior [29]. In anthropogenic winter smog regimes, such as the Upper Silesia region evaluated in this work, inorganic combustion aerosols dominate particle hygroscopicity. In contrast, in marine environments influenced by sea spray aerosols, arid zones dominated by mineral dust, or coastal regions subject to frequent fog, differing optical refractive indices, particle size distributions, and deliquescence thresholds will shift the non-linear response curves between ambient *RH*, temperature, and measured PM mass [11,47,48].

To systematically evaluate model portability and calibration stability under contrasting aerosol compositions, future research will focus on testing and fine-tuning these trained models across diverse environmental testbeds. In particular, upcoming field deployments are planned in different geographic locations, including the Baltic Sea coastal area, to assess model generalizability under marine aerosol conditions and high coastal relative humidity dynamics.

While this benchmark establishes that static non-linear models effectively correct relative humidity artifacts without the high computational overhead of deep recurrent networks, translating these findings into autonomous, field-deployed monitoring nodes represents an important next step. The practical implementation of software-based dehumidification on resource-constrained microcontrollers involves several key engineering and operational considerations.

A primary direction for future work is the direct C/C++ embedded implementation of the top-performing models, specifically sixth-degree Polynomial Regression and optimized Decision Trees, on edge hardware such as ESP8266 and ESP32 microcontrollers. Although offline Python benchmarking demonstrated sub-millisecond inference times, embedded field testing will allow us to profile real-time execution constraints, memory allocation (RAM and Flash utilization), and floating-point computation overhead directly on low-cost IoT hardware. Crucially, this deployment will include a detailed power consumption assessment. Physical heating elements used in hardware dehumidification represent a major energy drain, often making solar- or battery-powered operation infeasible. Quantifying the energy saved by replacing resistive heaters with lightweight software inference will highlight the sustainability and operational benefits of software-based calibration for dense monitoring networks. A second critical aspect concerns model generalizability across individual sensor units of the same type. Low-cost optical PM sensors often exhibit manufacturing tolerances, baseline drift, and unit-to-unit variations in laser diode intensity and airflow dynamics. Consequently, a key open question is whether a single universal software model can be deployed across an entire fleet of sensors, or whether each unit requires individual calibration. Future research will explore calibration transfer strategies across multiple identical sensors. Addressing on device power profiling and sensor-to-sensor transferability will bridge the gap between algorithmic benchmarking and scalable, energy-efficient air quality monitoring networks.

A key consideration in evaluating the presented software humidity compensation framework is the nature of the target benchmark. In this study, model training and performance evaluation were conducted using paired measurements from two co-located SDS011 optical sensors, where one unit operated with an active PTC heating element and the second unit remained unheated. Consequently, the target labels represent the readings of a physically dehumidified low-cost sensor rather than absolute, reference-grade atmospheric PM2.5 and PM10 concentrations from regulatory gravimetric or beta attenuation monitors. While heating the intake airflow effectively evaporates surface moisture and mitigates hygroscopic overestimation above 60% relative humidity, an SDS011 sensor, even when heated, remains subject to inherent optical sensor limitations, including particle size selectivity, refractive index assumptions, and fan flow variations. Therefore, the reported agreement metrics ($R^{2}$, RMSE, MAE) quantify how closely software algorithms can replicate the output of a hardware heating element, demonstrating that software compensation can functionally replace physical heaters on low-power IoT nodes. However, these metrics do not establish direct equivalence to standard regulatory reference methods. Future work will focus on co-locating both heated and software-dehumidified sensor nodes with certified reference instruments to evaluate absolute concentration accuracy and establish formal equivalency functions.

## 5. Conclusions

This study demonstrates that software-based humidity compensation provides an energy-efficient alternative to physical heating elements in low-cost optical particulate matter sensors under high-relative-humidity conditions. By evaluating six machine learning architectures against a co-located, physically heated sensor in Upper Silesia, Poland, we established that lightweight static non-linear models, particularly sixth-degree Polynomial Regression and Decision Trees, accurately reproduce heated sensor readings with R-squared values up to 95% for PM10 and 94.0% for PM2.5. Complex sequential architectures like LSTM yielded lower accuracy across all evaluated historical window lengths, confirming that instantaneous meteorological parameters drive operational humidity artifacts at ten-minute aggregation scales. With sub-millisecond inference latencies, static non-linear models enable real-time edge execution on low-cost microcontrollers, eliminating the high power consumption of resistive heaters and making battery- or solar-powered sensor nodes viable.

Several limitations and future directions guide the ongoing development of this approach. Currently, the models emulate the output of a physically heated low-cost sensor rather than an independent regulatory reference monitor, making future validation against certified reference instruments essential. Additionally, because training was conducted at a single site, model parameters reflect local conditions and will require local retraining when deployed in different climate zones or aerosol regimes. Upcoming field deployments are planned in contrasting environments, specifically including the Baltic Sea coastal area, to evaluate model transferability under marine aerosol conditions. Future work will also focus on porting top-performing models to optimized embedded C code for direct on-device execution, conducting power consumption profiling, and testing calibration transfer protocols across multi-unit sensor fleets.

## Figures and Tables

**Table 1:** Summary statistics of environmental and meteorological variables.

Variable	Mean	Std	Min	25%	75%	Max
PM2.5_heated_	14.62	14.52	0.18	4.68	19.96	411.96
PM10_heated_	28.41	31.79	0.57	7.88	38.14	910.85
PM2.5_unheated_	15.03	14.22	0.20	5.28	20.45	383.30
PM10_unheated_	30.88	33.24	0.60	8.97	41.75	942.65
Temperature (°C)	6.56	7.01	−10.91	1.74	11.03	27.36
Pressure (hPa)	992.06	9.09	959.72	986.78	998.51	1013.34
Humidity (%)	72.49	12.90	26.49	66.49	81.84	97.14

**Table 2:** Computational complexity and execution times across calibration models for PM_2.5_.

Model	Tuning	Time/Fit ^1^	Training Time ^2^	Inference Time ^3^
	Fits	[s]	[s]	[ms/sample]
Polynomial Reg. (d=6)	N/A	N/A	0.34	0.0011
Decision Tree	7490	0.004	8.23	0.0002
Support Vector Machine	494	1.9067	428.73	0.0003
Dense Neural Net (DNN)	250	10.973	5.68	0.0245
Long Short-Term Memory (LSTM)	100	148.957	122.52	0.1215

^1^ Average time per tuning evaluation fit. ^2^ Final model execution time on the entire training dataset. ^3^ Average prediction latency computed per single 10-min measurement sample.

## Data Availability

The datasets collected and analyzed during the current study are available in the RepOD, Repository Open Data, at https://doi.org/10.18150/1QFISN, accessed on 1 May 2026.

## Appendix A. Summary Table of Literature Evaluation Results

In this appendix, Table A1 provides a comprehensive overview of the 21 reviewed sources, detailing the studied low-cost sensors (LCSs), reference instrumentation, calibration methodology/models, and statistical performance metrics ($R^{2}$, MAE, RMSE/MSE, etc.).

**Table A1:** Summary of low-cost particulate matter (PM) sensor evaluation results across all 21 reviewed studies.

#	References and Method	Studied Low-Cost Sensor(s)	Reference Sensor	Analyzed Metrics	Main Conclusions
[5]	Hofman et al. (2021), Variational Graph Autoencoder (AVGAE) and Graph Random Forest (GRF)	SPS30 (Sensirion)	Regulatory air quality stations (RIVM)	$R^{2}$, MAE, RMSE	GRF seems to achieve slightly better accuracies, while the correlations are typically higher for AVGAE; results show that model performance still relies on the spatial representativity (spatial monitoring coverage).
[13]	Zimmerman et al. (2018), Random Forest (RF)	RAMP monitor (Alphasense CO, ${\mathrm{NO}}_{2}$, $O_{3}$, ${\mathrm{SO}}_{2}$ and NDIR ${\mathrm{CO}}_{2}$)	Mobile lab reference monitors and ACHD regulatory site	$R^{2}$, MAE, CvMAE	RF model applied to the RAMP low-cost sensor package can accurately characterize air pollution concentrations at the low levels typical of many urban areas in the United States and Europe.
[14]	Liu et al. (2021), stepwise regression model, the standard Multilayer Perceptron neural network and the SRA-MLP	Self-built miniature air quality detector (PM_2.5_, PM_10_, gases, *T*, *RH*, *P*)	National control point station	RMSE, MAE, MAPE, Accuracy	Using the SRA-MLP model to correct the data can increase the accuracy of the self-built point data by 42.5% to 86.5%.
[21]	Gryech et al. (2020), Multiple Linear Regression (MLR), Support Vector Regression (SVR), Random Forest (RF)	Nova PM SDS011 sensor (LILI-1/MoreAir system)	Alphasense OPC-N3	RMSE, $R^{2}$	Random Forest is shown to provide better performance than Support Vector Machine and Multiple Linear Regression.
[22]	Wang, Lung, & Liu (2020), Multiple Linear Regression (MLR), Support Vector Regression (SVR), and Random Forest regression (RFR)	Plantower PMS5003	Taiwan EPA stations (Met One BAM-1020, VEREWA-F701)	RMSE, $R^{2}$, MAE, RMSE	RFR was superior to MLR and SVR in its correction accuracy and computing efficiency.
[7]	Vajs et al. (2021), Linear Regression (LR), Artificial Neural Network (ANN), Random Forest (RF)	EkoNET sensing unit (Alphasense CO, ${\mathrm{NO}}_{2}$, Plantower PMS7003 PM_10_)	Automatic Monitoring Station run by the Serbian Environmental Protection Agency	$R^{2}$, RMSE	Incorporating ambient parameters (*T*, *RH*) yields stronger performance in non-linear models like RF. RF has shown better performances for NO2 and PM10 pollutants, while ANN was better for CO. Depending on the observed period, R2 is in the range from 0.927–0.970 for CO, 0.817–0.943 for NO2 and 0.731–0.891 for PM10.
[15]	Huang et al. (2022), Multiple Linear Regression (MLR), Random Forest (RF)	AirBeam2 sensors	TSI DustTrak DRX Aerosol Monitor 8533	$R^{2}$, RMSE, ME	Accuracy and bias of PM concentrations recorded by the AirBeam2 would be affected due to weather conditions (i.e., rainy days), environments with relatively high humidity, a high level of hygroscopic salts (i.e., seaside), and different aggregation levels of the temporal unit. The results also indicate that using RF models would generate better results than MLR models for the data collected on sunny days.
[26]	Vajs, Drajic, & Cica (2023), Random Forest (RF)	EkoNET hybrid sensor network (10 units; Plantower PMS7003 PM_10_, Alphasense ${\mathrm{NO}}_{2}$)	Automatic Monitoring Station run by the Serbian Environmental Protection Agency (SEPA)	RMSE, Correlation coefficient (*r*)	Improvement of up to 0.35/0.14 for the Pearson correlation coefficient and a reduction of 6.82 $\mu {g/m}^{3}$/20.56 $\mu {g/m}^{3}$ for the RMSE, for NO2 and PM10.
[23]	Villanueva et al. (2023), Univariate Linear Regression (ULR), Multivariate Linear Regression (MLR), Nearest-Neighbors Regression (KNN), Support Vector Regression (SVR), AdaBoost Regression, Gradient Boosting Regression (GB), Random Forest Regression (RF), and Extra Trees Regression (ET).	IQAir devices with light-scattering laser photo sensors and AirBeam with Plantower PMS7003	Teledyne API T640 Mass Monitor	$R^{2}$, RMSE	Proposed strategy combining pre-fit calibration models on an ensemble of sensors together with a reduced period (4 days) of field data from the candidate sensor can provide performance similar to that of the best fitted monosensor model adjusted with field data from that sensor over a ten-times-longer period.
[16]	El Mghouchi et al. (2024), Artificial Neural Network (ANN), Support Vector Machine (SVM), Decision Tree (DT), Gaussian Process Regression (GR), and Linear Regression (LR).	PM Smoggie and A3 820002C3	Manufacturer chamber/EU-accredited laboratory reference	$R^{2}$, MAPE,	Feature Selection–DT combined model proved to be the best model for predicting the PM concentrations with very high correlation and approximations. The humidity was the least significant variable in the PM concentrations, while the best accuracy was found by combining pressure with temperature.
[28]	Sooriyaarachchi et al. (2024), Convergent Cross Mapping (CCM) combined with ML methods	Alphasense OPC-N3 optical particle counter	GRIMM Laser Aerosol Spectrometer and Dust Monitor Model 1.109 (Germany)	MSE, $R^{2}$	Convergent Cross Mapping (CCM) reduces model complexity while increasing generalizability.
[17]	Smith et al. (2025), Decision Tree Regressor (DTR), Random Forest (RF), K-Nearest Neighbor, XGBRegressor, Support Vector Regression (SVR), Simple Neural Network (SNN), Deep Neural Network (DNN), Long Short Term Memory (LSTM) neural network, Recurrent Neural Network (RNN), Ordinary Least Square Regression (OLS), and Least Absolute Shrinkage and Selection Operator (Lasso) regression	PurpleAir PA-II PM_2.5_ sensors (PMS*003 series)	US EPA regulatory monitoring stations	$R^{2}$, RMSE	LSTM models excelled, with high R2 scores of 0.856 and 0.857 and low RMSEs of 4.25 $\mu {g/m}^{3}$ and 4.26 $\mu {g/m}^{3}$, respectively; LSTM models may be too slow (1.5 h) or computation-intensive; tree-boosted models including XGBoost (0.7612, 5.377 $\mu {g/m}^{3}$) and Random Forest (RF) (0.7632, 5.366 $\mu {g/m}^{3}$) offered good performance with shorter training times (<1 min).
[18]	Balagopal et al. (2025), Random Forest, Ensemble Bagging, Light Gradient Boosting Machine, Extreme Gradient Boosting, Decision Tree, Neural Network, K-Nearest Neighbors, Linear Regression, Ridge Regression and Stacking Models	LoRaWAN IoT air quality monitors (PM_1.0_, PM_2.5_, PM_4.0_, PM_10.0_)	Palas Fidas Frog research-grade spectrometer	$R^{2}$	The Stacking Regressor demonstrated near-research-grade accuracy in predicting PM concentrations across various size fractions, outperforming traditional tree-based models.
[3]	Mehmet Taştan (2025), Decision Tree (DT), Linear Regression (LR), Random Forest (RF), k-Nearest Neighbors (kNN), AdaBoost (AB), Gradient Boosting (GB), Support Vector Machines (SVMs), and Stochastic Gradient Descent (SGD).	IoT air quality monitor (ESP8266 + PM_2.5_ sensor, ${\mathrm{CO}}_{2}$, *T*, *RH*)	Dienmern DM72b (Langdeli Technology Co., Ltd., Shenzhen, China)	$R^{2}$, RMSE, MAE, MAPE	GB and kNN achieved the highest accuracy. For CO2 sensor calibration, GB achieved R2 = 0.970, RMSE = 0.442, and MAE = 0.282, providing the lowest error rates. For the PM2.5 sensor, kNN delivered the most successful results, with R2 = 0.970, RMSE = 2.123, and MAE = 0.842. Additionally, for temperature and humidity sensors, GB demonstrated the highest accuracy with the lowest error values (R2 = 0.976, RMSE = 2.284).
[4]	Tasmurzayev et al. (2025), Logistic Regression (LR), Decision Tree (DT), XGBoost, Random Forest (RT)	Tynys mobile IoT sensor (Winsen ZPHS01B); ${\mathrm{CO}}_{2}$, PM_2.5_, PM_10_)	Qingping Air Monitor	Accuracy, Precision, Recall, F1-Score	The XGBoost model achieved the best performance, attaining 91.25% accuracy in classifying pollution levels across the three transport modes.
[27]	Li et al. (2025), Random Forest (RF), XGBoost, and CatBoost as base models; Ridge Regression as meta model	SDL307 low-cost PM_2.5_ sensors (4 units)	National Environmental Monitoring Station in Hankou Jiangtan, Wuhan City	$R^{2}$, RMSE, MAE, MAPE	Constructed model shows good prediction ability in 5-fold cross-validation, with an overall R2 of 0.93 and a root mean square error (RMSE) of 3.09 $\mu {g/m}^{3}$.
[19]	Gómez, Rodríguez, & Ferro (2026), Linear Regression (LR), Generalized Regression (GR), Decision Tree (DT), KNN, XGBoost, and Artificial Neural Network (ANN)	Sensirion SPS30 PM_2.5_ and PM_10_	Teledyne T640X particle monitor (Bogotá)	$R^{2}$, RMSE, MAE	For both PM10 and PM2.5, the performance evaluation showed the superiority of the Random Forest and XGBoost models. For PM10, Random Forest achieved the lowest RMSE ≈ 31 and the highest R2 > 93.6%, while XGBoost yielded the lowest MAE ≈ 0.021 and an R2 above 92.9%. For PM2.5, Random Forest obtained the lowest RMSE ≈ 2.99 and MAE 0.019, both with R2 > 96.5%.
[20]	Mamani-Suclla et al. (2026), 44 different ML models	PMS5003	IQAir platform (publicly available air quality data)	$R^{2}$, RMSE, MAE	Traditional statistical models exhibit significant limitations in capturing the non-linear dynamics of particulate matter concentrations. In contrast, tree-based and boosting algorithms achieved superior predictive performance, with R2 values at or above 0.90 for both target variables. Deep learning architectures produced competitive results but did not consistently surpass boosting models. The stacking approach combining a Ridge meta-learner with K-Fold cross-validation emerged as the most effective configuration, achieving an R2 of 0.9019 for PM2.5 and an R2 of 0.9170 for PM10 prediction.
[24]	Owczarek, Rogulski, & Czechowski (2020), regression models	Low-cost PM_10_ candidate devices (CM1, CM2; optical sensors)	WIOŚ station in Nowy Sącz (gravimetric reference method, RM)	Adjusted $R^{2}$, $R^{2}$, Relative Uncertainty	The best model: generalized linear regression models using PM10 concentration values from the tested devices, wind speed and humidity.
[25]	Minxing Si, Ying Xiong, Shan Du, and Ke Du (2020), simple linear regression (SLR), Multiple Linear Regression (MLR), XGBoost and a feedforward neural network (NN)	Plantower PMS 5003	Hybrid Ambient Real-time Particulate (SHARP) monitor at the Calgary Varsity air monitoring station	Pearson *r*, MAE, RMSE	Feedforward NN is a promising method to address the poor performance of low-cost sensors for PM2.5 monitoring.

## Appendix B. Optimal Model Hyperparameters

The hyperparameters of the Decision Tree optimized model are as follows:

ccp_alpha:0.00083,
criterion:absolute_error,
max_depth:20,
max_features:4,
min_samples_leaf:7,
min_samples_split:53,
splitter: ’best’. 
		

The hyperparameters of the best obtained SVM model are as follows:

C:3, degree:1,
epsilon:0.005,
gamma:auto,
tol:0.001
		

**Table A2:** Best neural network model architectures for PM_2.5_ and PM_10_ targets.

Model	PM_2.5_ Target Architecture	PM_10_ Target Architecture
DNN	4-layer DNN: Input(4) → Normalization → Dense(128, 128, 128, 96) with BatchNorm + ReLU → Dense(1); HeUniform, L2 decay.	4-layer DNN: Input(4) → Normalization → Dense(112, 80, 64, 48) with BatchNorm + ReLU → Dense(1); HeUniform, L2 decay.
LSTM	3-stacked LSTM: Input(30, 4) → Normalization → LSTM(128) → Dropout(0.3) → LSTM(32) → LSTM(32) → Dense(1); L2 decay.	5-stacked LSTM: Input(30, 4) → LSTM(128) → LSTM(80) → Dropout(0.4) → LSTM(80) → LSTM(64) → Dropout(0.4) → LSTM(96) → Dropout(0.4) → Dense(1); L2 decay.
